# Class IIa HDACs in myelination

**DOI:** 10.18632/aging.101443

**Published:** 2018-05-05

**Authors:** Sergio Velasco-Aviles, Jose A. Gomez-Sanchez, Hugo Cabedo

**Affiliations:** 1 Instituto de Neurociencias, Universidad Miguel Hernández-CSIC and Instituto de Investigación Sanitaria y Biomédica de Alicante (ISABIAL)-FISABIO, Alicante, Spain

**Keywords:** myelin development, Schwann cells, cAMP, epigenetics, histone deacetylases

Myelin is a highly specialized plasma membrane that wraps around and insulates axons to allow rapid saltatory nerve conduction. Schwann cells produce the myelin sheath in the peripheral nervous system, whereas a distinctly different cell (the oligodendrocyte) myelinates axons in the central nervous system.

Although myelin is largely absent from neonatal peripheral nerves, larger axons are already sorted and wrapped in a 1:1 ratio by individual Schwann cells spaced along their length. Later these cells will up-regulate genes encoding for the enzymes that synthesize lipids, enabling expansion of the plasma membrane to form the myelin sheath, as well as a set of structural proteins that compact and stabilize the myelin. This genetic program is determined by the transcription factor *Egr2 (krox20)* and involves downregulation of the “negative regulator” of myelination c*-Jun* [1].

It has been demonstrated that a rise in cAMP induced by the G protein-coupled receptor 126 (Gpr126) controls this Schwann cell phenotypic transition *in vivo* [2]. Similarly, differentiation is also observed *in vitro* when Schwann cells are exposed to relatively high concentrations of cAMP analogs or inducers. In contrast a modest increase in cAMP potentiates the mitogenic effects of some trophic factors but has no impact on differentiation. cAMP activates PKA and EPAC with similar potency raising the question of how it might impact differentially on Schwann cells to control the myelination program [3].

Gomis-Coloma et al [4] have recently shown that the cAMP-induced shuttling of class IIa histone deacetylases (HDACs) into the nucleus of Schwann cells is necessary and sufficient to downregulate *c-Jun* and activate the myelin transcriptional program. Translocation is in part mediated by phosphorylation by PKA. Significantly only cAMP-concentrations that induce Schwann cell differentiation translocate class IIa HDACs into the nucleus.

HDACs are classified into four main families: classes I, IIa, IIb and IV. At variance with other families, class IIa HDACs (4,5,7 and 9) have no prominent deacetylase activity, since a pivotal tyrosine in the catalytic site is mutated to histidine [5]. Instead they work mainly as co-repressors of transcription factors such as Mef2. However, Gomis-Coloma et al found that mutations in HDAC4 that block binding to Mef2 do not impair its capacity to downregulate *c-Jun* in Schwann cells.

How then HDAC4 can downregulate *c-Jun* and induce Schwann cell differentiation? It has been elegantly demonstrated by Fischel et al [5] that HDAC4 recruits HDAC3 (a class I HDAC) by forming a multiprotein complex with the NCoR1/SMRT co-repressor. Thus, HDAC4 can recruit a *bona fide* deacetylase, HADC3, to repress gene expression. Gomis-Coloma et al show that in Schwann cells, HDAC4 indeed binds to the multiprotein complex NCoR1/SMRT-HDAC3. Moreover, mutations that block the interaction of HDAC4 with this complex impair its ability to downregulate *c-Jun*. Finally, these authors demonstrate that the capacity of cAMP to downregulate *c-Jun* in Schwann cells disappears when the deacetylase activity is inhibited with triscostatin A.

How is HDAC4 recruited to the *c-Jun* promoter? Because HDACs do not bind DNA directly, they need to interact with transcription factors to be localized to the target promoters. An obvious candidate is Mef2, but as mentioned before, Gomis-Coloma et al have shown that binding to Mef2 is not necessary to downregulate *c-Jun*. An alternative candidate is c-Jun itself, as an AP-1 binding site is located in the *c-Jun* promoter, constituting a positive feedback loop that is probably involved in the rapid expression of this early gene. Further experiments are needed to clarify this point.

Is *c-Jun* the only gene repressed by HDAC4 in Schwann cells? Importantly, the authors have also shown that HDAC4 is recruited to the promoters of *Runx2* and *GDNF*, suggesting that class IIa HDACs can bind and repress directly other genes of non-myelin forming and repair Schwann cells. Suggestively c-Jun also binds to these promoters, supporting a role of this transcription factor in the recruitment of HDAC4. Whether it binds the promoters of other negative regulators of myelination (such as *Sox2* and *Ednrb*) needs to be explored.

Using conditional knock out mice, Gomis-Coloma et al also show that the coordinated removal of two class IIa HDACs (HDAC4 and HDAC5) from Schwann cells delays myelin development. Although it supports a central role of these HDACs in the formation of myelin *in vivo,* several questions remain to be answered. Why is it necessary to remove two class IIa HDACs to observe a phenotype? and why does a total arrest of myelination not occur? Although encoded by different genes, all class IIa HDACs share molecular profiles and regulate equivalent genetic programs in other cells. For instance, it is necessary to remove at least four alleles of some of the skeletal muscle expressed class IIa HDACs to observe a change in muscle fiber phenotype [6]. Thus, a compensatory effect within the members of the family seems to be working in muscle, and is therefore likely to be operative in Schwann cells as well. Importantly Schwann cells also express HDAC7 [4], which could partially compensate for the absence of HDAC4 and HDAC5. Further *in vivo* data will be necessary to clarify these questions.

**Figure 1:**
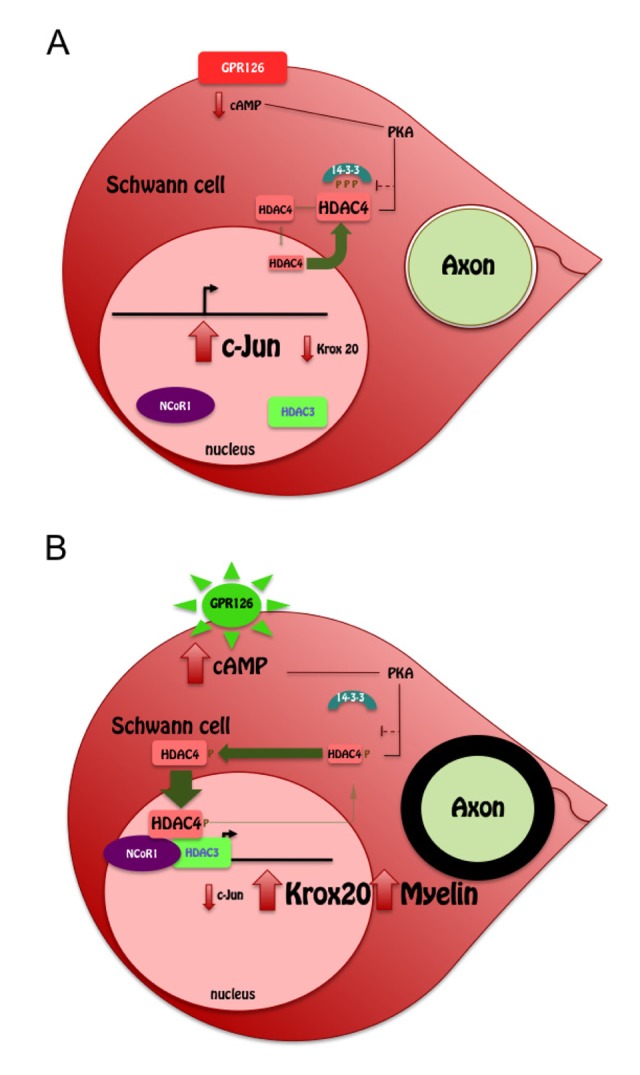
(**A**) At low cAMP concentrations, 14-3-3 sequesters HDAC4 in the cytosol allowing *c-Jun* expression (**B**)**.** Gpr126 activation raises intracellular cAMP levels activating PKA. This shuttles HDAC4 into the nucleus, where it binds to the promoter of *c-Jun* recruiting the NCoR1/HDAC3 complex to block *c-Jun* expression. Then *Krox20* expression drives myelin development.

It was recently shown that Zeb2 represses the negative regulators of myelination *Sox2* and *Ednrb* allowing myelination to proceed [7]. This indicates that the repression of “negative regulators” is pivotal for proper myelination. Gomis-Coloma et al provide evidence showing that the repression of another negative regulator of myelination (*c-Jun*) is also relevant for myelin development. Importantly, the repressor in this case (a class IIa HDAC) is translocated and activated by intracellular cAMP, which links Gpr126-mediated signaling with the myelin transcriptional program in Schwann cells (Figure 1).
